# Visible-Light-Curable Solvent-Free Acrylic Pressure-Sensitive Adhesives via Photoredox-Mediated Radical Polymerization

**DOI:** 10.3390/molecules26020385

**Published:** 2021-01-13

**Authors:** Jong-Ho Back, Yonghwan Kwon, Hyun-Joong Kim, Youngchang Yu, Wonjoo Lee, Min Sang Kwon

**Affiliations:** 1 Center for Advanced Specialty Chemicals, Korea Research Institute of Chemical Technology, Ulsan 44412, Korea; beak1231@krict.re.kr; 2 Department of Materials Science and Engineering, Seoul National University, Seoul 08826, Korea; yhkwon@unist.ac.kr; 3 Department of Materials Science and Engineering, Ulsan National Institute of Science and Technology (UNIST), Ulsan 44919, Korea; 4 Department of Agriculture, Forestry and Bioresources, Research Institute of Agriculture and Life Sciences, College of Agriculture and Life Science, Seoul National University, Seoul 08826, Korea; hjokim@snu.ac.kr

**Keywords:** acrylic pressure-sensitive adhesive, visible-light curing, photocatalysis, α-haloester

## Abstract

Owing to their excellent properties, such as transparency, resistance to oxidation, and high adhesivity, acrylic pressure-sensitive adhesives (PSAs) are widely used. Recently, solvent-free acrylic PSAs, which are typically prepared via photopolymerization, have attracted increasing attention because of the current strict environmental regulations. UV light is commonly used as an excitation source for photopolymerization, whereas visible light, which is safer for humans, is rarely utilized. In this study, we prepared solvent-free acrylic PSAs via visible light-driven photoredox-mediated radical polymerization. Three α-haloesters were used as additives to overcome critical shortcomings, such as the previously reported low film curing rate and poor transparency observed during additive-free photocatalytic polymerization. The film curing rate was greatly increased in the presence of α-haloesters, which lowered the photocatalyst loadings and, hence, improved the film transparency. These results confirmed that our method could be widely used to prepare general-purpose solvent-free PSAs—in particular, optically clear adhesives for electronics.

## 1. Introduction

Pressure-sensitive adhesives (PSAs) are polymers that are adhesive at room temperature and can be easily attached to objects under low applied pressure. PSAs are widely used in our daily lives as label materials, double-sided tapes and adhesives, foam tapes, and electrical tapes. Recently, special-grade PSAs have been used in different industries, including the electronics, automotive, and medical industries [1]. PSAs are typically classified into acrylic, rubber, and silicone according to their components [1,2,3]. Of these, acrylic PSAs present excellent adhesive properties, transparency, resistance to oxidation, and nonyellowing [1]. The acrylic PSA market reached US $8.84 billion in 2017 [4]. In particular, the solvent-free acrylic PSA market has grown because of the government regulations for low-volatile organic compound emissions and the elimination of the drying process, reducing manufacturing costs [1,5,6,7].

**Scheme 1:**
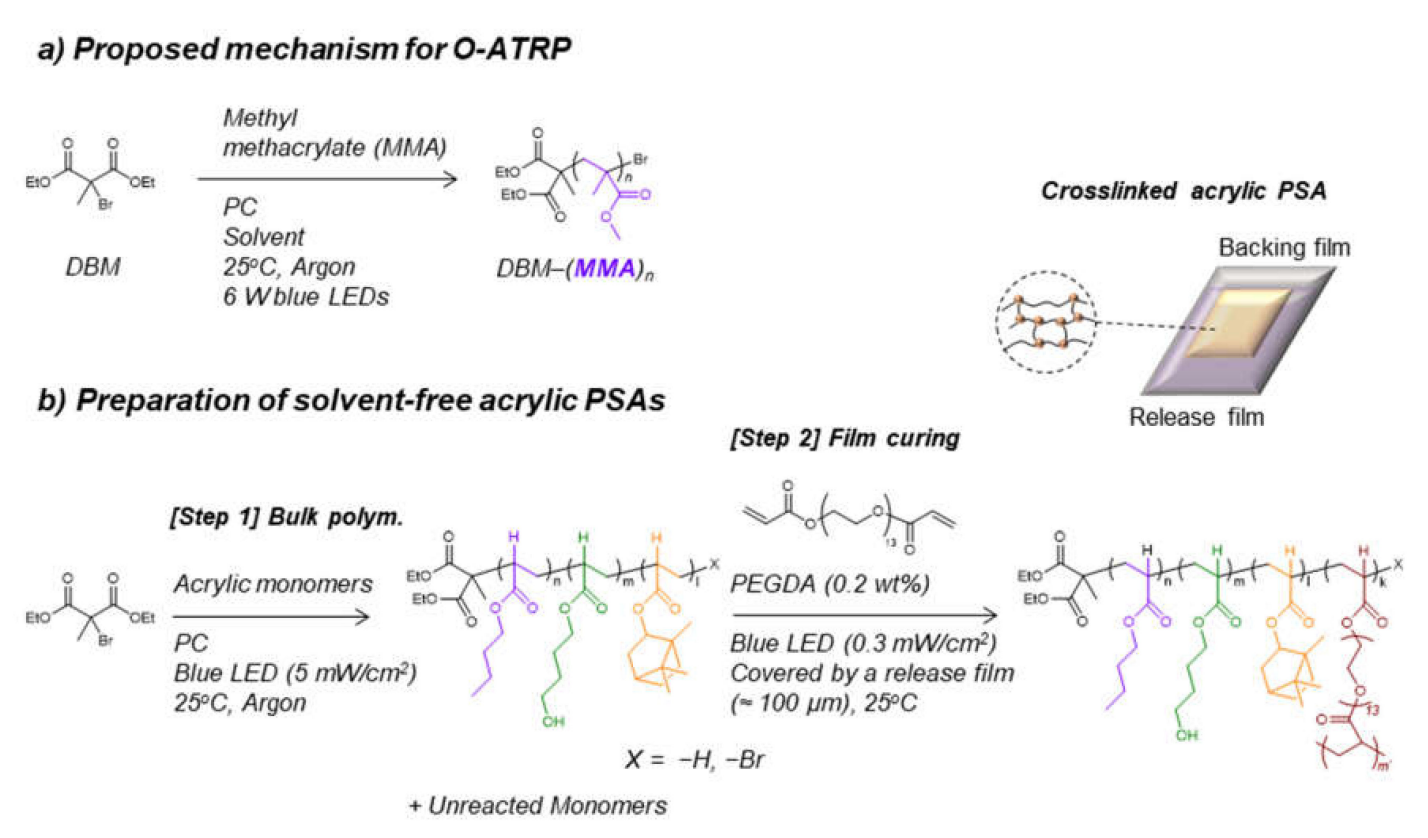
(**a**) Proposed mechanism for diethyl 2-bromo-2-methylmalonate (DBM)-initiated O-atom transfer radical polymerization (O-ATRP). Detail mechanism is presented in the Supplementary Materials. (**b**) Manufacturing process of solvent-free acrylic pressure-sensitive adhesives (PSAs) via photocatalytic free radical polymerization. Here, Et, PC, and PEGDA denote ethyl, photocatalyst, and poly(ethylene glycol) diacrylate, respectively.

Solvent-free acrylic PSAs are typically produced via UV curing using a two-step process that involves bulk polymerization and film curing (Scheme 1) [6,7]. Photoinitiators (PIs) are commonly used during bulk polymerization to prepare viscous acrylic prepolymers using different acrylic monomers whose compositions significantly affect PSA properties. After bulk polymerization, the crosslinking agent and PI are added to the prepared acrylic prepolymer, and the mixture is used to form a film. The PSA film is subsequently cured with UV light, and the film surface is typically covered with a release film to avoid oxygen inhibition [8].

Photocatalyzed radical polymerization is a powerful light-driven polymerization method [9,10,11]. Since most photocatalysts (PCs) exhibit sufficient visible-light absorption, photocatalyzed polymerization can be conducted under low-energy visible light irradiation conditions using sunlight and light-emitting diodes (LEDs) as light sources [12,13], which are safer for humans than UV light. Visible light-absorbing PCs have been widely used for numerous light-driven photocatalyzed radical polymerization reactions, including photocatalyzed free radical polymerization [14,15,16], photoinduced electron/energy transfer–reversible addition-fragmentation chain transfer [17,18,19,20], and photoredox-mediated atom transfer radical polymerization (ATRP) [21,22,23,24], which can used for a wide range of applications, such as 3D/4D printing [25,26,27,28], biosystems [28,29,30], hydrogels [28,29,31,32,33], dental materials [34,35,36], and coatings [37,38].

Recently, we prepared solvent-free acrylic PSAs via photoredox-mediated free radical polymerization using low PC loadings (as low as 50 ppm) under visible light (455 nm) irradiation conditions [39]. In this previous study, we used *N*-vinyl-based monomers as a key component to increase the bulk polymerization and film curing rates by facilitating electron transfer during initiation. However, the film curing rate was still lower than that of PI radical polymerization. Moreover, the transparency of the prepared film in the visible region was rather low, because the residual PCs could absorb visible light. Therefore, a further increase in the polymerization rate at low PC loadings is required to overcome the aforementioned drawbacks.

In this study, the bulk polymerization and film curing rates in the presence of α-haloesters were much higher than those of previously reported photocatalyzed free radical polymerization [39], even at extremely low PC loadings, and therefore, the prepared acrylic PSA films presented high transparency. Kinetic studies on bulk polymerization and film curing revealed that the optimum conditions consisted of a catalyst loading of 10 ppm and an α-haloester concentration of 0.1 mol%. Gel content and adhesion performance analyses and UV–Vis transmittance experiments confirmed that the prepared PSA films could be widely used as general-purpose solvent-free PSAs, including optically clear adhesives for electronics. Fast film curing, which was comparable to that of current photoinitiated polymerization reactions, and high transparency of the prepared PSAs suggest that our strategy could replace conventional PI radical polymerization.

## 2. Results and Discussion

### 2.1. Strategy

As in our previous research [39], we used 2,4,5,6-tetrakis(diphenylamino)-1,3-benzenedicarbonitrile (4DP-IPN) as the PC, owing to its excellent catalytic performance, which is ascribed to its strong visible-light absorption, suitable redox potential, high stability, and efficient generation of triplet excitons [24]. In addition, the high solubility of 4DP-IPN for a variety of monomers maximized its utility.

**Scheme 2:**
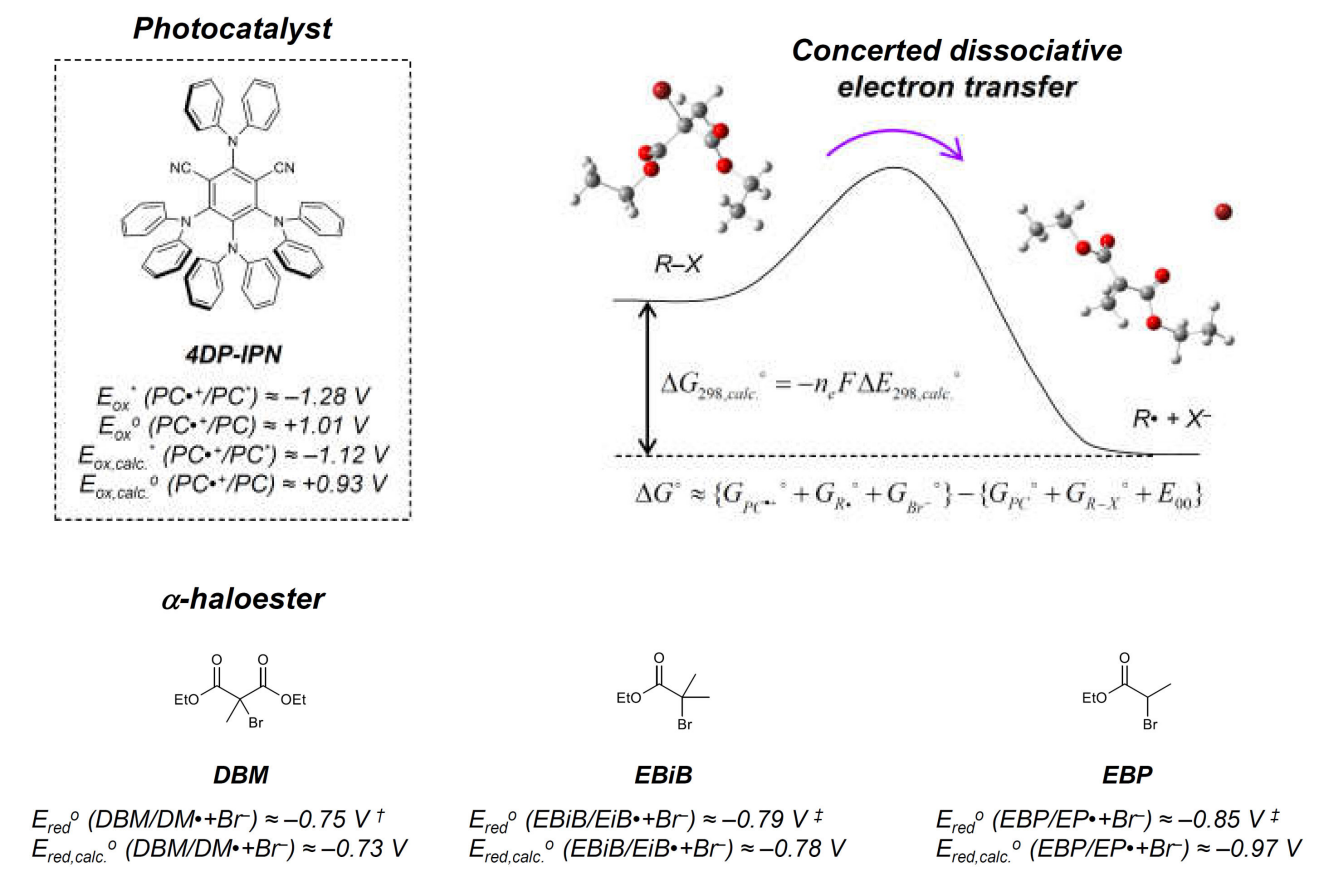
Chemical structures of the 2,4,5,6-tetrakis (diphenylamino)-1,3-benzenedicarbonitrile (4DP-IPN) photocatalyst and α-haloesters (diethyl 2-bromo-2-methylmalonate (DBM), ethyl 2-bromopropionate (EBP), and ethyl α-bromoisobutyrate (EBiB); the experimental/computational redox potentials are also indicated. ^†^ Value was derived from reference [40]. ^‡^ Value was derived from reference [41]. The time-dependent (TD) density functional theory (DFT) calculations were performed with the B3LYP functional and 6–311G * basis set in ethyl acetate using the polarizable continuum model (PCM). The detailed (TD) DFT method, molecular orbital (MO) diagrams, and MO topologies are described in the Supplementary Materials.

To increase the polymerization and film curing rates, we selected α-haloesters as an additive. In fact, α-haloesters are commonly used as an initiator in photoredox-mediated ATRP, because they can generate radical species through dissociative electron transfer with PCs [24]. Three α-haloesters with suitable ground-state reduction potentials (*E_red_*) were selected, and it was expected that the electron transfer would be favorable, considering that the excited-state oxidation potential (E_ox_ *) of 4DP-IPN was –1.28 V vs. the saturated calomel electrode (SCE, Scheme 2). As presented in Scheme 2, the quantum chemical calculation and reported experimental data also supported our arguments [40,41]. The calculated ground-state reduction potentials (*E_red,cal_*) of the selected α-haloesters were much less negative than the E_ox_ * of 4DP-IPN, which are also well in accordance with the experimental reduction potentials (*E_red_*). This demonstrated that the transfer of electrons from the excited state of 4DP-IPN to each α-haloester is exothermic based on the Rehm-Weller equation [42], and thus, the electron transfer kinetics between 4DP-IPN and the selected α-haloesters are expected to be favorable considering the Marcus—Sevéant theory, which describes the kinetics of concerted dissociative electron transfer [42,43].

### 2.2. Preparation of Solvent-Free Acrylic PSAs

#### 2.2.1. Bulk Polymerization

We first investigated the effects of α-haloesters on the bulk polymerization reaction performed to prepare a prepolymer mixture. The polymerization of n-butyl acrylate (BA) with 4-hydroxybutyl acrylate (HBA) and isobornyl acrylate (IBOA) was conducted as a control experiment using a PI loading of 340 ppm under an inert Ar atmosphere and 365-nm UV irradiation (Entry 3, Table 1). The aforementioned monomers are commonly used to prepare solvent-free acrylic PSAs [1]. The control experiment delivered poly(BA-*co*-HBA-*co*-IBOA) with a conversion (α) of 7.4% and a molecular weight (M_n_) of 873 kg/mol after only 15 s of UV irradiation; moreover, the prepared prepolymer mixture was viscous enough to be coated as a film. As negative control experiments, polymerization was performed in the absence of 4DP-IPN and α-haloesters (Entry 1, Table 1) and in the absence of 4DP-IPN (Entry 2, Table 1) under 455-nm blue LED irradiation. As expected, no polymers were synthesized in either experiment, which suggested that both the PC and an α-haloester were necessary for efficient initiation.

Entry 4 in Table 1 presents the results of the photocatalytic radical polymerization of acrylic monomers in the absence of an α-haloester using 455-nm blue LED irradiation. The monomer composition and atmospheric condition were the same as those used during the photoinitiated experiment. A long irradiation time of over 600 s was required to obtain a proper prepolymer mixture (α = 11.2% and M_n_ = 418 kg/mol). When diethyl 2-bromo-2-methylmalonate (DBM) was used as the α-haloester, the polymerization time to achieve a similar conversion (α = 11.7%) was significantly shortened (10 s, Entry 5, Table 1), and this was ascribed to the efficient radical initiation of DBM [16]. The polymerization rate significantly increased as the concentration of DBM increased in the presence of 50 and 10 ppm of 4DP-IPN (Table S1), and the optimum DBM concentration was determined to be 0.1 mol% (Entries 5 and 6, Table 1). The initiation of photocatalytic radical polymerization was affected by the ratio of α-haloester-to-4DP-IPN, and the optimized 4DP-IPN loading was determined to be 10 ppm (Entry 6, Table 1) rather than 50 (Entry 5, Table 1) and 2 ppm (Entry S5, Table S1). The amount of DBM consumed at the 4DP-IPN loading of 2 ppm was small (0.2%) compared with those consumed when the 4DP-IPN loadings were 10 and 50 ppm (8.1% and 11.8%, respectively; Figure S2). These results indicated that the initiation was inefficient at the 4DP-IPN loading of 2 ppm and DBM concentration of 0.1 mol% (Entry S5, Table S1). Furthermore, two other α-haloesters—namely, ethyl 2-bromopropionate (EBP) and ethyl α-bromoisobutyrate (EBiB)—were used. The polymerization rate in the presence of EBiB (Entry 8, Table 1, α = 7.45% after 100 s) was higher than that in the presence of EBP (Entry 7, Table 1, α = 7.76% after 180 s). However, the polymerization kinetics in the presence of EBP and EBiB were slower than that in the presence of DBM, which indicated that the polymerization kinetics could be accelerated by the strong driving force for electron transfer from the excited state of 4DP-IPN to the α-haloester, facilitating a more favorable electron transfer. As this trend was in agreement with the *E_red,cal_* values, EBP, the α-haloester with the most negative *E_red,cal_* (−0.97 V vs. SCE), exhibited the slowest polymerization kinetics of all the α-haloesters in this study.

#### 2.2.2. Film Curing

The acrylic prepolymer was mixed with poly (ethylene glycol) diacrylate (M_n_ = 700 g/mol, Sigma-Aldrich, St. Louis, MO, USA), and the resulting mixture was coated on a polyethylene terephthalate film; the final thickness of the PSA layer was approximately 100 μm. The film was subsequently covered with a release film to prevent oxygen inhibition (Figure S3). PCs or α-haloesters were not used for film curing.

The film was cured under 455-nm blue LED irradiation. The curing rate was estimated by measuring the conversion of monomers, which was determined using Fourier-transform infrared spectroscopy. The conversion of the monomers was calculated from the decrease in the C=C peak intensity (1660–1600 cm^−1^) with respect to the C=O peak intensity (1760–1660 cm^−1^), which remained unchanged during film curing [44].

**Figure 1:**
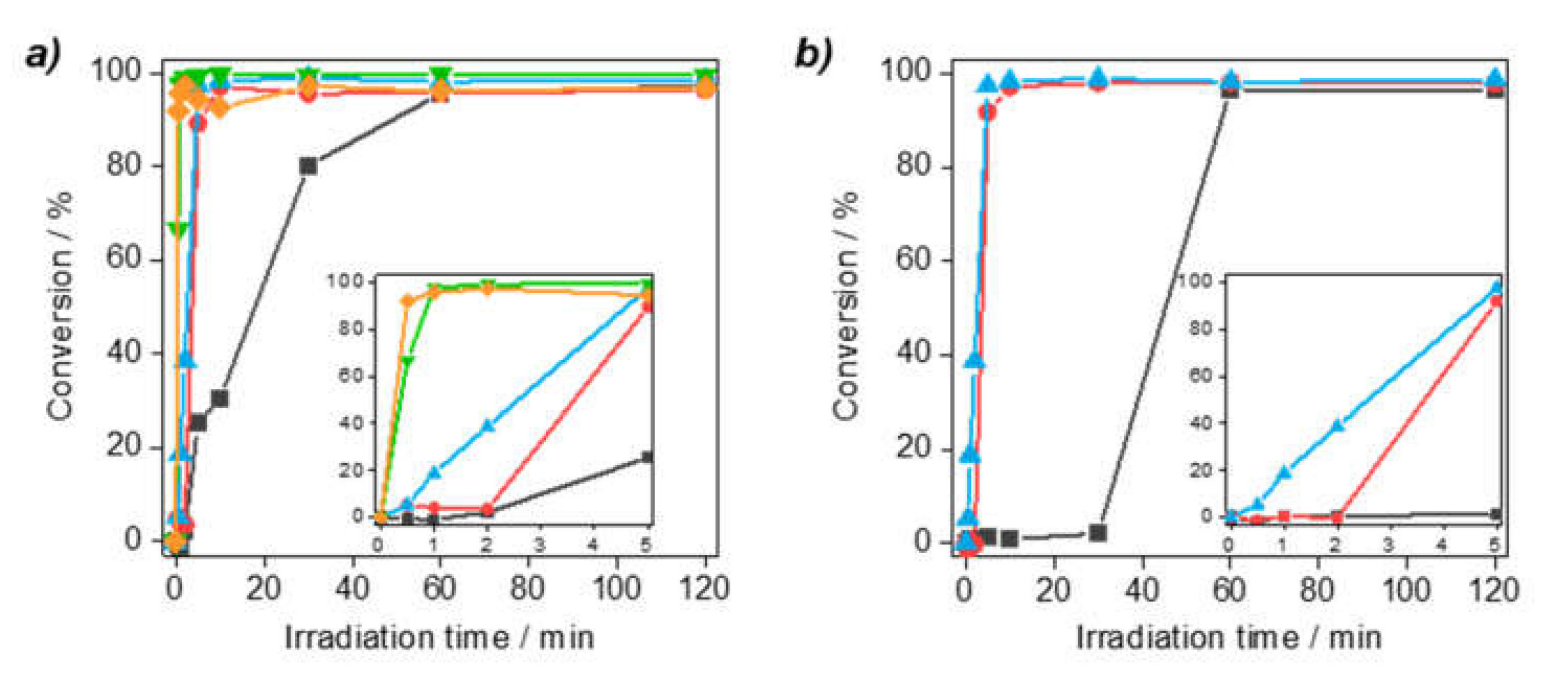
Conversion dependency of the irradiation time during film curing: (**a**) at a set loading of 2,4,5,6-tetrakis(diphenylamino)-1,3-benzenedicarbonitrile (4-DP-IPN) of 50 ppm and different concentrations of diethyl 2-bromo-2-methylmalonate (DBM) (gray squares: 0 mol%, red circles: 0.05 mol%, sky blue triangles: 0.1 mol%, green inverted triangles: 0.5 mol%, and orange diamonds: 5 mol%) and (**b**) at a set concentration of DBM of 0.1 mol% and different loadings of 4-DP-IPN (gray squares: 2 ppm, red circles: 10 ppm, and sky blue triangles: 50 ppm).

As illustrated in Figure 1a, the film curing rate was significantly higher in the presence of DBM than in its absence. A very high conversion of approximately 98% was achieved within five min of 455-nm blue LED irradiation even at a low DBM concentration of 0.1 mol% and a 4DP-IPN loading of 10 ppm. This conversion was comparable to that of conventional UV curing systems, which typically required six min for film curing [8]. The large amounts of unused DBM and Br-terminated polymers generated during bulk polymerization facilitated the photoinduced electron transfer from 4DP-IPN, which resulted in the significant increase in the film curing rate. To further optimize the PC loading, we analyzed the effect of the PC loading on the film curing rate using different 4DP-IPN loadings and a set concentration of DBM (0.1 mol%). The film curing rate was very low at a low 4DP-IPN loading of 2 ppm, whereas the curing rates at the 4DP-IPN loadings of 10 and 50 ppm were similar and much higher (Figure 1b). Considering the bulk polymerization and film curing results, we determined that the optimum 4DP-IPN loading and DBM concentration were 10 ppm and 0.1 mol%, respectively. Therefore, we concluded that the addition of a small amount of DBM to the reaction system caused the bulk polymerization and film curing rates to increase more than six times (the irradiation time, to achieve complete film conversion, decreased from 60 to 10 min) even at a low 4DP-IPN loading of 10 ppm, which was five times lower than that reported in our previous paper (50 ppm) [39].

### 2.3. Characterization of Solvent-Free Acrylic PSAs

#### 2.3.1. Gel Content

**Figure 2:**
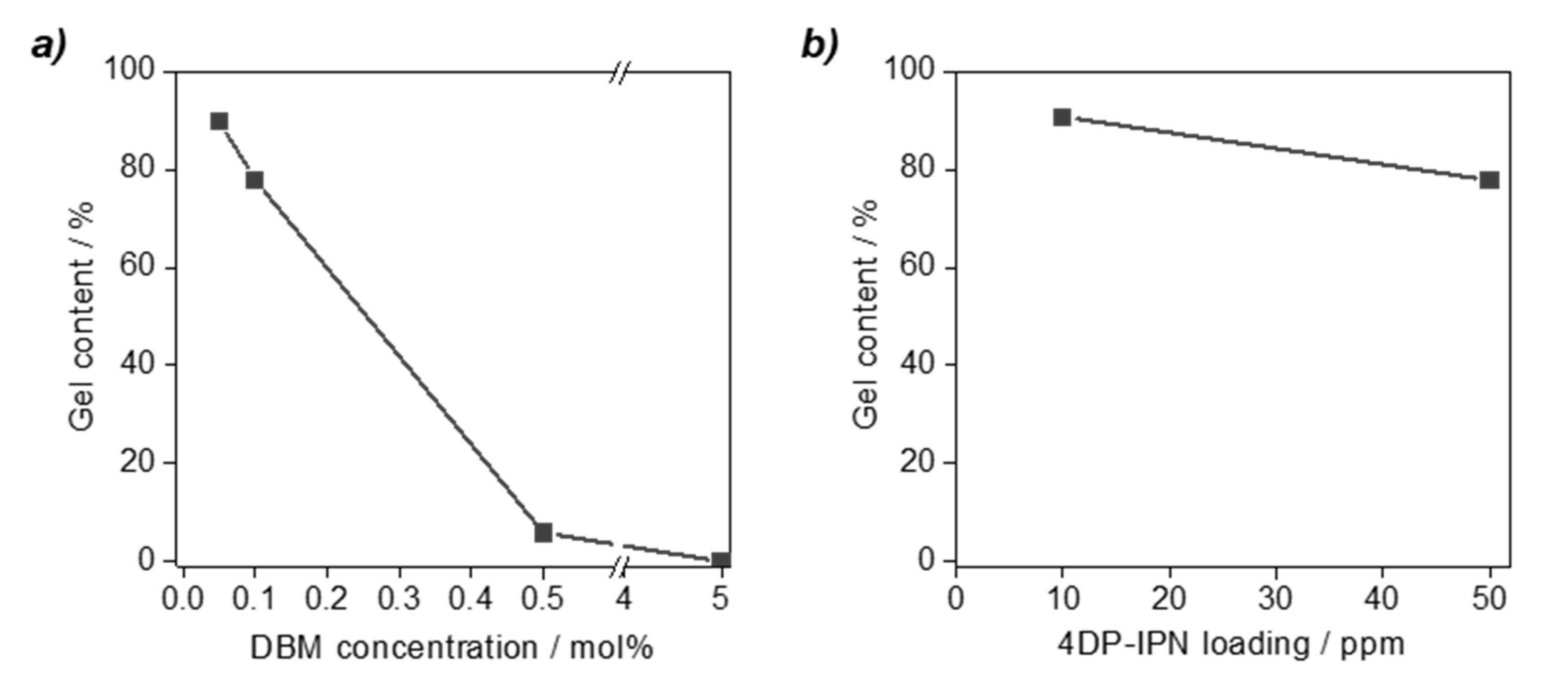
Gel content of the cured acrylic pressure-sensitive adhesives prepared using different: (**a**) diethyl 2-bromo-2-methylmalonate (DBM) concentrations (the 2,4,5,6-tetrakis(diphenylamino)-1,3-benzenedicarbonitrile (4DP-IPN) loading and curing time were 50 ppm and 30 min, respectively) and (**b**) 4DP-IPN loadings (the DBM concentration and curing time were 0.1 mol% and 30 min, respectively).

PSA film curing was further analyzed by measuring the gel contents of the prepared films under two different conditions: a set 4DP-IPN loading of 50 ppm and a set DBM concentration of 0.1 mol% (Figure 2a,b, respectively). At the set 4DP-IPN loading of 50 ppm, the gel content decreased with the increasing DBM concentration, which might be attributed to the decrease in chain length with increasing initiator content. When a DBM concentration of 5 mol% was used, a large amount of DBM (92.1%), which could initiate the curing reaction, remained in the prepolymer mixture (Figure S4). This caused a decrease in molecular weight and an insufficient crosslinking density of the cured PSA [45,46]. In other words, when the concentration of DBM exceeded the optimal level (0.1 mol%) for bulk polymerization and film curing, a sufficiently crosslinked network could not be built. Furthermore, at the set DBM concentration of 0.1 mol%, the cured PSA formed a sufficiently crosslinked network and, therefore, maintained a high gel content, even if the 4DP-IPN loading was reduced from 50 to 10 ppm, which was the optimal 4DP-IPN loading for fast film curing.

#### 2.3.2. Transparency

**Figure 3:**
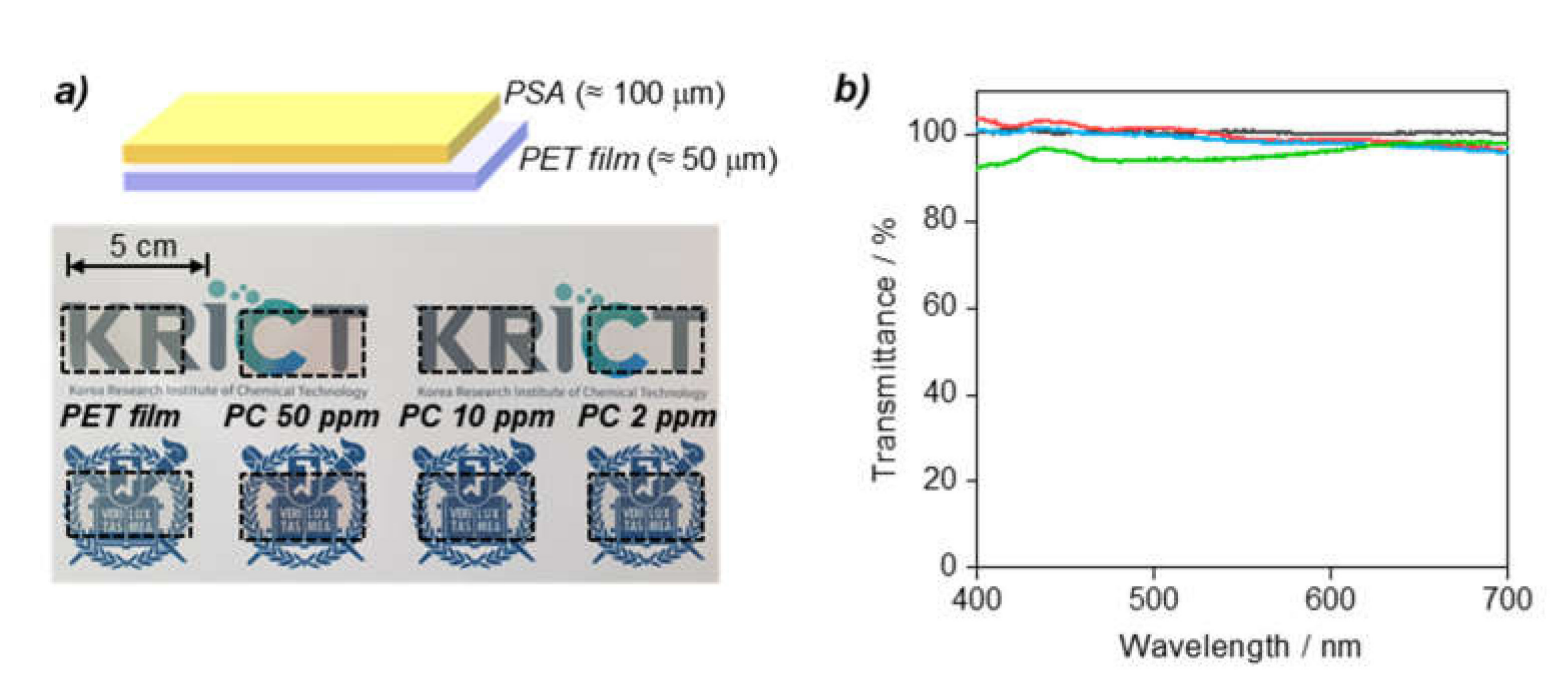
Transparency of the cured acrylic pressure-sensitive adhesive (PSA): (**a**) vertical structure (top) and transparency (bottom) of the PSA. (**b**) UV–Vis spectrum of the PSA obtained at a diethyl 2-bromo-2-methylmalonate concentration of 0.1 mol%. The gray, red, sky blue, and green lines represent the support polyethylene terephthalate (PET) film without a PSA layer and PSA films prepared using 2,4,5,6-tetrakis(diphenylamino)-1,3-benzenedicarbonitrile (4DP-IPN) loadings of 2, 10, and 50 ppm, respectively. The curing time was 30 min for all samples, except for the PSA prepared at a 4DP-IPN loading of 2 ppm, which was cured for 2 h for sufficient conversion.

To assess the feasibility of the cured PSAs for optical applications, their transmittance was evaluated using UV–Vis spectroscopy (Figure 3b). 4DP-IPN presented remarkable UV–Vis absorption (300–500 nm, Figure S6); therefore, the optical transparency of the PSAs was significantly increased from 94.5% to 99.1% by decreasing the 4DP-IPN loading from 50 to 2 ppm. The transparency of the cured PSA obtained using a 4DP-IPN loading of 10 ppm was 98.5%, which was similar to those of the PSAs obtained using a 4DP-IPN loading of 2 ppm (99.1%) and a PI (98.2%, Figure S5). The transparencies of the cured PSAs obtained using 4DP-IPN loadings of 2 and 10 ppm were high, so that these PSAs could be used as optically clear adhesives with a light transmission of 99% (3M^TM^ Optically Clear Adhesives 8211, 8212, 8213, 8214, and 8215).

#### 2.3.3. Adhesion Performances

**Figure 4:**
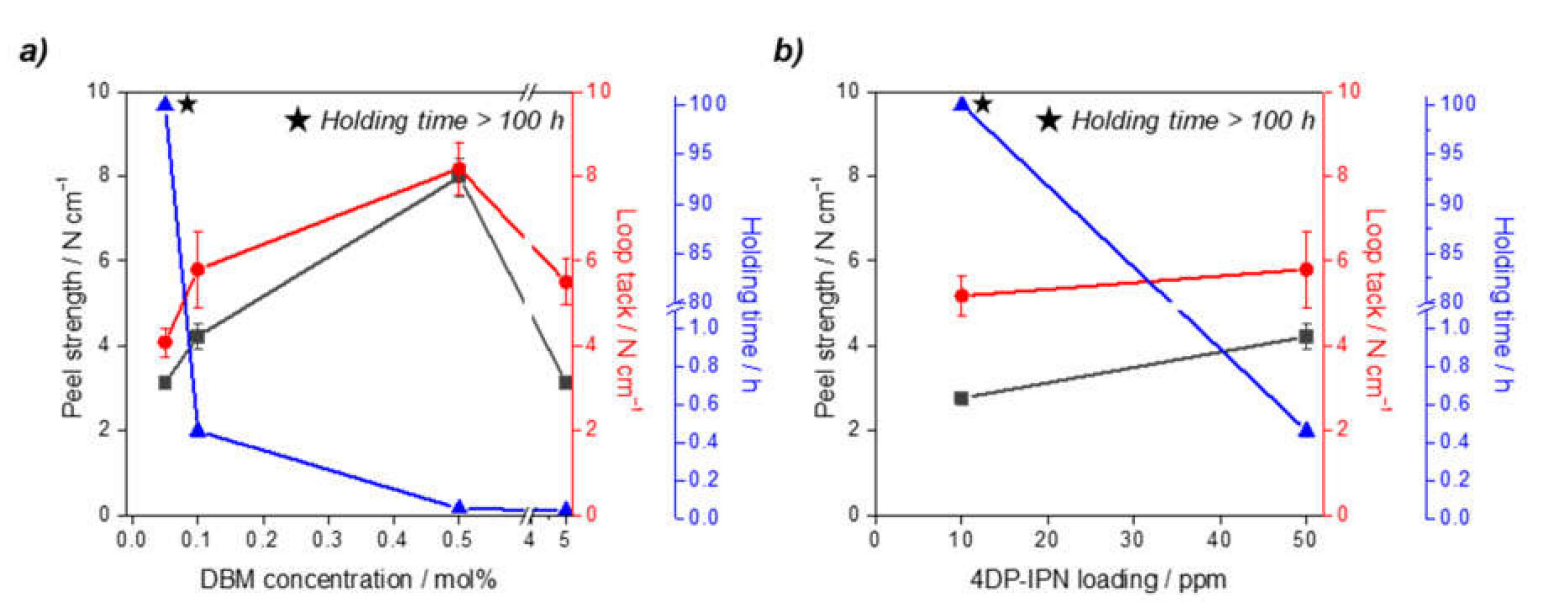
Adhesion performances of cured acrylic pressure-sensitive adhesives obtained using different: (**a**) diethyl 2-bromo-2-methylmalonate (DBM) concentrations (the 2,4,5,6-tetrakis(diphenylamino)-1,3-benzenedicarbonitrile (4DP-IPN) loading and curing time were 50 ppm and 30 min, respectively) and (**b**) 4DP-IPN loadings (the DBM concentration and curing time were 0.1 mol% and 30 min, respectively). The gray squares, red circles, and blue triangles represent the peel strength, loop tack, and holding time, respectively.

The adhesion performance of the cured PSAs—namely, peel strength, loop tack, and holding time—was evaluated to confirm whether they could be used as conventional acrylic PSAs. As the concentration of DBM increased at a set 4DP-IPN loading of 50 ppm, the peel strength and loop tack of the prepared PSAs decreased after increasing, whereas the holding time steadily decreased (Figure 4a). This could be attributed to a decrease in the gel content of the PSAs, because a low gel content could cause improvement on wetting but decrease in creep resistance [2]. The sharp decline in the adhesion performance of the PSA prepared using a DBM concentration of 5 mol% could be caused by the significant decrease in the molecular weight of the PSA owing to the excess amount of the initiator. When the 4DP-IPN loading was lowered from 50 ppm to 10 ppm at a set DBM concentration of 0.1 mol%, the holding time of the prepared PSA was greatly increased, whereas the peel strength and loop tack were mostly unchanged (Figure 4b). These findings could be explained by the increase in the gel content of the PSAs with decreasing 4DP-IPN loading (Figure 2b).

The peel strength (2.8–8.0 N cm^−1^) and loop tack (4.1–8.2 N cm^−1^) of the prepared PSAs indicated that the PSAs could be used as general acrylic PSAs and optically clear adhesives [8,47,48,49]. In particular, the PSA obtained using a DBM concentration of 0.1 mol% and a 4DP-IPN loading of 10 ppm presented both excellent creep resistance (holding time > 100 h) and transparency (98.5%), which rendered it the most suitable PSA as an optically clear adhesive for electronics. In conclusion, the polymerization (bulk polymerization and film curing) and characterization (transmittance and adhesion performances) results indicated that the optimum conditions for preparing a PSA that could replace conventional UV-curable optically clear adhesives consisted of a DBM concentration of 0.1 mol% and a 4DP-IPN loading of 10 ppm.

## 3. Materials and Methods

### 3.1. Materials

Butyl acrylate (BA, 99%, Sigma-Aldrich, St. Louis, MO, USA), isobornyl acrylate (IBOA, technical grade, Sigma-Aldrich, St. Louis, MO, USA), and 4-hydroxybutyl acrylate (HBA, 97%, TCI, Tokyo, Japan) were used as acrylate monomers. Inhibitor in the monomer was removed by alumina basic. 4DP-IPN was used as the visible-light photocatalyst, and the synthesis method is described in the references [20,24]. UV/Vis spectrum and photoluminescence intensity were shown in Figure S6. Diethyl 2-bromo-2-methylmalonate (DBM, 98%, Sigma-Aldrich, St. Louis, MO, USA) and poly(ethylene glycol) diacrylate (PEGDA, M_n_: 700 g/mol, Sigma-Aldrich, St. Louis, MO, USA) were used as a radical initiator and a crosslinker, respectively.

### 3.2. Preparation of the Solvent-Free Acrylic PSA

#### 3.2.1. Bulk Polymerization

The mixture of monomer, photocatalyst, and α-haloester was stirred at a speed of 300 rpm and degassed by Ar purging for 30 min. The composition of the acrylate monomer was set as (BA):(IBOA):(HBA) = 80:10:10, and the total mass of monomer was fixed as 5 g. Bulk polymerization was conducted through blue LED irradiation (455 nm, 5 mW/cm^2^) at room temperature (Figure S3). Irradiation time was set as 10 s, and the conversion at the bulk polymerization was gravimetrically evaluated. For Entry 3 in Table 1, an UV photoinitiator (Irgacure 184, 0.05 wt%) and UV lamp (365 nm, 3 mW/cm^2^) were used. A representative sample (Entry 6, Table 1) was characterized by ^1^ H-NMR and differential scanning calorimetry, as shown in Figure S11.

#### 3.2.2. Film Curing

A crosslinker (PEGDA, 0.2 wt%) was added into the acrylic prepolymer that was prepared by bulk polymerization. The mixture was stirred for 5 min and applied in a film form onto the 50 μm of PET film. A release film covered the surface of the applied PSA in order to prevent oxygen inhibition during film curing (Figure S3). The thickness of the acrylic PSA layer was set as 100 μm. The film curing was conducted under blue LED irradiation (455nm, 0.3 mW/cm^2^) at room temperature. The conversion at the film curing was estimated by the consumption of C=C bonds, which was characterized using Fourier-transform infrared spectroscopy (FTIR, Nicolet 6700, Thermo Fisher Scientific, Waltham, MA, USA) in ATR mode, as shown in Figure S7.

### 3.3. Characterization of the Solvent-Free Acrylic PSA

#### 3.3.1. Gel Content

When the cured PSA was dissolved in toluene, crosslinked polymers were not dissolved but swollen, while the linear polymer was fully dissolved. In order to evaluate the contents of the crosslinked polymers, i.e., gel contents, the mixture of PSA and toluene was filtered by a stainless-steel mesh (#200). The crosslinked polymers remained on the mesh, but the uncrosslinked polymers passed through the stainless-steel mesh. The gel content was calculated as follows (Equation (1), Figure S8):

(1) $$\mathrm{Gel}\;\mathrm{content}\;\left(\%\right)=\;\frac{W_{r}}{W_{t}}\times 100$$

where *W_r_* and *W_t_* represent the weight of the residue (crosslinked polymers) and total weight of the cured PSA film, respectively.

#### 3.3.2. Adhesive Properties

For the 180° peel test, the cured PSA with 1 cm of width was attached to the substrate (SUS 304) by a constant force (2-kg roller twice), followed by being kept at room temperature for 24 h. The peel strength was evaluated by a universal testing machine (UTM, LS1, AMETEK, Berwyn, PA, USA) with 5 mm/s of constant machine speed. The average debonding force, i.e., peel strength was calculated in the range from 20% to 80% of a working distance (Figure S9a). The peel test was repeated for four times for each entry.

The loop tack test was conducted with a string form of the cured PSA (length: 15 cm and width: 1 cm). The string-formed PSA approached the substrate (SUS 304), and thus, the PSA touched the surface of the substrate. After contact, the PSA was immediately detached from the substrate with 5 mm/s of constant speed (Figure S9b). The loop tack test and 180° peel test were conducted at room temperature. The loop tack test was repeated for four times for each entry.

The creep resistance of the cured PSA was evaluated through a holding test. The cured PSA was attached on the substrate (SUS 304) by a constant force (2-kg roller twice), and the adhesion area was fixed as 15 mm × 15 mm. The holding test was performed 1 h after the attachment, and the holding time, i.e., the time that the PSA endured the weight of 1 kg at 50 °C, was recorded (Figure S9c). The holding test was conducted by single experiment.

#### 3.3.3. UV/Vis Spectroscopy

A test sample for UV/Vis spectroscopy was prepared, as shown in Figure 3a. Transmittance was evaluated in the range of wavelength from 400 nm to 700 nm using an UV/Vis spectrometer (Evolution 600, Thermo Fisher Scientific, Waltham, MA, USA). The backing film was located on the reference cell, and the baseline was calibrated by measuring the transmittance of the backing film without a PSA layer.

#### 3.3.4. Size Exclusion Chromatography (SEC)

The molecular weight of the polymer was characterized by size exclusion chromatography (1260 Infinity ‖ LC, Agilent technologies, Santa Clara, CA, USA; chamber temperature: 40 °C; detector: reflex index detector; and column: Shodex^TM^ KF-G, 602, 604, and 605). Tetrahydrofuran (99.9%, HPLC grade, Samchun Chemicals, Pohang, Korea) was used as the eluent with a constant flow rate (0.5 mL min^−1^), and the results were calibrated using polystyrene standards (Figure S12).

## 4. Conclusions

In this study, the drawbacks of the visible light-driven photocatalytic polymerization of solvent-free acrylic PSA (i.e., low curing rate and poor transparency) were successfully addressed. The addition of an α-haloester to the reaction system significantly increased the bulk polymerization and film curing rates to levels similar to those of conventional UV-curable acrylic PSAs. This lowered the PC content and, therefore, improved the optical transparency. Time-dependent density-functional theory calculations confirmed that the kinetic enhancement was driven by an increase in the initiation rate owing to the efficient electron transfer between the α-haloester and PC. Furthermore, we determined the optimum conditions (a DBM concentration of 0.1 mol% and a 4DP-IPN loading of 10 ppm) that increased the film curing rate and facilitated the synthesis of a PSA with excellent transparency, which was suitable as an optically clear adhesive for electronics. We believe that the PSAs prepared in this study could substitute conventional UV-curable optically clear adhesives that require PIs. Since the adhesion performance at optimum conditions was limited by the single composition of the acrylate monomers we used, in future studies, different compositions should be considered to construct optically clear adhesives with a wide adhesion performance range.

## Figures and Tables

**Table 1:** Bulk polymerization results; α = gravimetrically determined conversion of bulk polymerization. Here, M denotes the monomer mixture, M_n_ and Ð denote the number of the average molecular weight and dispersity, respectively, which were determined using size-exclusion chromatography, and DBM, EBP, EBiB, and 4DP-IPN denote diethyl 2-bromo-2-methylmalonate, ethyl 2-bromopropionate, ethyl α-bromoisobutyrate, and 2,4,5,6-tetrakis(diphenylamino)-1,3-benzenedicarbonitrile, respectively.

Entry	α-Haloester	(M) ^a^:(4DP-IPN): (α-Haloester)	Time (s)	α (%)	M_n_ (kg/mol)	Ð
1	-	100:0:0	>8 h	No separable polymers		
2	DBM	100:0:0.1	>8 h	No separable polymers		
3 ^b^	-	100:0.034:0	15	7.42	873	1.68
4	-	100:0.005:0	600	11.2	418	2.34
5	DBM	100:0.005:0.1	10	11.7	289	2.51
6	DBM	100:0.001:0.1	10	7.68	512	2.35
7	EBP	100:0.005:0.1	180	7.76	749	1.83
8	EBiB	100:0.005:0.1	100	7.45	514	2.20

^a^ Comprising n-butyl acrylate (BA), isobornyl acrylate (IBOA), and 4-hydroxybutyl acrylate (HBA) (mole ratio was set as follows: (BA):(IBOA):(HBA) = 80:10:10).^b^ A photoinitiator was used instead of the 4DP-IPN photocatalyst.

## Data Availability

The data presented in this study are available in article and supplementary materials.

## Supplementary Materials

The following are available online. Figure S1: Nonconsumable PC (UV/Vis spectrum). Figure S2: Remaining DBM% after bulk polymerization (GC). Figure S3: Manufacturing process of solvent-free acrylic PSA. Figure S4: Results with 50-ppm 4DP-IPN and 5 mol% DBM. Figure S5: UV/Vis spectrum of PSA prepared through an UV photoinitiator. Figure S6: UV/Vis spectrum and PL intensity of 4DP-IPN. Figure S7: Calculation of conversion at film curing (FTIR). Figure S8: Method for the evaluation of the gel contents. Figure S9: Method for the evaluation of the adhesive properties. Figure S10: Lap shear strength. Figure S11: Characterizations of a representative sample. Figure S12: Calibration curve of size-exclusion chromatography. Figure S13: DFT calculations. Table S1: Bulk polymerization (supporting). Table S2: Reproducibility. References from Supplementary materials [50,51,52,53].

## References

[1] Satas D. (1989). Handbook of Pressure Sensitive Adhesive Technology.

[2] Benedek I., Heymans L.J. (1997). Pressure-Sensitive Adhesives Technology.

[3] Creton C. (2003). Pressure-sensitive adhesives: An introductory course. MRS Bull..

[4] (2017). Acrylic Adhesives Market by Type (Acrylic Polymer Emulsion, Cyanoacrylic, Methacrylic, UV Curable Acrylic), Application (Paper & Packaging, Construction, Transportation, Medical, Consumer, Electronics), Technology, and Region—Global Forecast to 2022.

[5] Scholz K., Hagenweiler K. (1972). Process for Preparing Solvent-Free Pressure-Sensitive Adhesive from a Polyisocyanate, a Polyoxyalkylated Diol or Polyol and a Tackifier. U.S. Patent.

[6] Czech Z., Milker R. (2003). Solvent-free radiation-curable polyacrylate pressure-sensitive adhesive systems. J. Appl. Polym. Sci..

[7] Glennon A.E. (1981). Pressure Sensitive Adhesives. U.S. Patent.

[8] Baek S.-S., Hwang S.-H. (2017). Preparation of biomass-based transparent pressure sensitive adhesives for optically clear adhesive and their adhesion performance. Eur. Polym. J..

[9] Dadashi-Silab S., Doran S., Yagci Y. (2016). Photoinduced electron transfer reactions for macromolecular syntheses. Chem. Rev..

[10] Corrigan N., Shanmugam S., Xu J., Boyer C. (2016). Photocatalysis in organic and polymer synthesis. Chem. Soc. Rev..

[11] Lee Y., Kwon M.S. (2020). Emerging organic photoredox catalysts for organic transformations. Eur. J. Org. Chem..

[12] Konkolewicz D., Schröder K., Buback J., Bernhard S., Matyjaszewski K. (2012). Visible light and sunlight photoinduced ATRP with ppm of Cu catalyst. ACS Macro Lett..

[13] Ciftci M., Tasdelen M.A., Yagci Y. (2014). Sunlight induced atom transfer radical polymerization by using dimanganese decacarbonyl. Polym. Chem..

[14] Xiao P., Zhang J., Dumur F., Tehfe M.A., Morlet-Savary F., Graff B., Gigmes D., Fouassier J.P., Lalevee J. (2015). Visible light sensitive photoinitiating systems: Recent progress in cationic and radical photopolymerization reactions under soft conditions. Prog. Polym. Sci..

[15] Lalevée J., Tehfe M.-A., Dumur F., Gigmes D., Blanchard N., Morlet-Savary F., Fouassier J.P. (2012). Iridium photocatalysts in free radical photopolymerization under visible lights. ACS Macro Lett..

[16] Zhang G., Song I.Y., Ahn K.H., Park T., Choi W. (2011). Free radical polymerization initiated and controlled by visible light photocatalysis at ambient temperature. Macromolecules.

[17] Xu J., Jung K., Atme A., Shanmugam S., Boyer C. (2014). A robust and versatile photoinduced living polymerization of conjugated and unconjugated monomers and its oxygen tolerance. J. Am. Chem. Soc..

[18] McKenzie T.G., Fu Q., Uchiyama M., Satoh K., Xu J., Boyer C., Kamigaito M., Qiao G.G. (2016). Beyond traditional RAFT: Alternative activation of thiocarbonylthio compounds for controlled polymerization. Adv. Sci..

[19] Chen M., MacLeod M.J., Johnson J.A. (2015). Visible-light-controlled living radical polymerization from a trithiocarbonate iniferter mediated by an organic photoredox catalyst. ACS Macro Lett..

[20] Song Y., Kim Y., Noh Y., Singh V.K., Behera S.K., Abudulimu A., Chung K., Wannemacher R., Gierschner J., Lüer L. (2019). Organic Photocatalyst for ppm-Level Visible-Light-Driven Reversible Addition–Fragmentation Chain-Transfer (RAFT) Polymerization with Excellent Oxygen Tolerance. Macromolecules.

[21] Fors B.P., Hawker C.J. (2012). Control of a living radical polymerization of methacrylates by light. Angew. Chem..

[22] Theriot J.C., Lim C.-H., Yang H., Ryan M.D., Musgrave C.B., Miyake G.M. (2016). Organocatalyzed atom transfer radical polymerization driven by visible light. Science.

[23] Pearson R.M., Lim C.-H., McCarthy B.G., Musgrave C.B., Miyake G.M. (2016). Organocatalyzed atom transfer radical polymerization using N-aryl phenoxazines as photoredox catalysts. J. Am. Chem. Soc..

[24] Singh V.K., Yu C., Badgujar S., Kim Y., Kwon Y., Kim D., Lee J., Akhter T., Thangavel G., Park L.S. (2018). Highly efficient organic photocatalysts discovered via a computer-aided-design strategy for visible-light-driven atom transfer radical polymerization. Nat. Catal..

[25] Al Mousawi A., Kermagoret A., Versace D.-L., Toufaily J., Hamieh T., Graff B., Dumur F., Gigmes D., Fouassier J.P., Lalevée J. (2017). Copper photoredox catalysts for polymerization upon near UV or visible light: Structure/reactivity/efficiency relationships and use in LED projector 3D printing resins. Polym. Chem..

[26] Al Mousawi A., Lara D.M., Noirbent G., Dumur F., Toufaily J., Hamieh T., Bui T.-T., Goubard F., Graff B., Gigmes D. (2017). Carbazole derivatives with thermally activated delayed fluorescence property as photoinitiators/photoredox catalysts for LED 3D printing technology. Macromolecules.

[27] Zhang Z., Corrigan N., Bagheri A., Jin J., Boyer C. (2019). A versatile 3D and 4D printing system through photocontrolled RAFT polymerization. Angew. Chem..

[28] Wang Z., Kumar H., Tian Z., Jin X., Holzman J.F., Menard F., Kim K. (2018). Visible light photoinitiation of cell-adhesive gelatin methacryloyl hydrogels for stereolithography 3D bioprinting. ACS Appl. Mater. Interfaces.

[29] Jeon E.Y., Hwang B.H., Yang Y.J., Kim B.J., Choi B.-H., Jung G.Y., Cha H.J. (2015). Rapidly light-activated surgical protein glue inspired by mussel adhesion and insect structural crosslinking. Biomaterials.

[30] Charron P.N., Fenn S.L., Poniz A., Oldinski R.A. (2016). Mechanical properties and failure analysis of visible light crosslinked alginate-based tissue sealants. J. Mech. Behav. Biomed. Mater..

[31] Aguirre-Soto A., Kim S., Kaastrup K., Sikes H.D. (2019). On the role of N-vinylpyrrolidone in the aqueous radical-initiated copolymerization with PEGDA mediated by eosin Y in the presence of O2. Polym. Chem..

[32] Sawhney A.S., Pathak C.P., Hubbell J.A. (1993). Interfacial photopolymerization of poly (ethylene glycol)-based hydrogels upon alginate-poly (l-lysine) microcapsules for enhanced biocompatibility. Biomaterials.

[33] Avens H.J., Bowman C.N. (2009). Mechanism of cyclic dye regeneration during eosin-sensitized photoinitiation in the presence of polymerization inhibitors. J. Polym. Sci. Part A Polym. Chem..

[34] Dressano D., Salvador M.V., Oliveira M.T., Marchi G.M., Fronza B.M., Hadis M., Palin W.M., Lima A.F. (2020). Chemistry of novel and contemporary resin-based dental adhesives. J. Mech. Behav. Biomed. Mater..

[35] Kamoun E.A., Winkel A., Eisenburger M., Menzel H. (2016). Carboxylated camphorquinone as visible-light photoinitiator for biomedical application: Synthesis, characterization, and application. Arab. J. Chem..

[36] Ge X., Ye Q., Song L., Misra A., Spencer P. (2015). Visible-Light Initiated Free-Radical/Cationic Ring-Opening Hybrid Photopolymerization of Methacrylate/Epoxy: Polymerization Kinetics, Crosslinking Structure, and Dynamic Mechanical Properties. Macromol. Chem. Phys..

[37] Allen N.S. (1996). Photoinitiators for UV and visible curing of coatings: Mechanisms and properties. J. Photochem. Photobiol. A.

[38] Bose S., Bogner R.H. (2010). Solventless visible light-curable coating: I. Critical formulation and processing parameters. Int. J. Pharm..

[39] Back J.-H., Kwon Y., Roldao J., Yu Y., Kim H.-J., Gierschner J., Lee W., Kwon M.S. (2020). Solvent-free acrylic pressure-sensitive adhesives via a visible-light driven photocatalytic radical polymerization without additives. Green Chem..

[40] Buss B.L., Lim C.H., Miyake G.M. (2020). Dimethyl Dihydroacridines as Photocatalysts in Organocatalyzed Atom Transfer Radical Polymerization of Acrylate Monomers. Angew. Chem..

[41] Inesi A., Rampazzo L., Zeppa A. (1981). Polarographic and voltammetric reduction of some aliphatic α-bromoesters. J. Electroanal. Chem. Interfacial Electrochem..

[42] Romero N.A., Nicewicz D.A. (2016). Organic photoredox catalysis. Chem. Rev..

[43] Saveant J.M. (1987). A simple model for the kinetics of dissociative electron transfer in polar solvents. Application to the homogeneous and heterogeneous reduction of alkyl halides. J. Am. Chem. Soc..

[44] Dietz J.E., Elliott B.J., Peppas N.A. (1995). Real-time attenuated total reflectance-fourier transform infrared spectroscopy to monitor multiacrylate polymerization reactions. Macromolecules.

[45] Zhao T., Zheng Y., Poly J., Wang W. (2013). Controlled multi-vinyl monomer homopolymerization through vinyl oligomer combination as a universal approach to hyperbranched architectures. Nat. Commun..

[46] Zhao T., Zhang H., Zhou D., Gao Y., Dong Y., Greiser U., Tai H., Wang W. (2015). Water soluble hyperbranched polymers from controlled radical homopolymerization of PEG diacrylate. RSC Adv..

[47] Sulley G.S., Gregory G.L., Chen T.T., Carrodeguas L.P., Trott G., Santmarti A., Lee K.-Y., Terrill N.J., Williams C.K. (2020). Switchable Catalysis Improves the Properties of CO2-Derived Polymers: Poly (cyclohexene carbonate-b-ε-decalactone-b-cyclohexene carbonate) Adhesives, Elastomers, and Toughened Plastics. J. Am. Chem. Soc..

[48] Beharaj A., Ekladious I., Grinstaff M.W. (2019). Poly (Alkyl Glycidate Carbonate) s as Degradable Pressure-Sensitive Adhesives. Angew. Chem..

[49] Park C.-H., Lee S.-J., Lee T.-H., Kim H.-J. (2015). Characterization of an acrylic polymer under hygrothermal aging as an optically clear adhesive for touch screen panels. Int. J. Adhes. Adhes..

[50] Harper T., Slegeris R., Pramudya I., Chung H. (2017). Single-Phase Photo-Cross-Linkable Bioinspired Adhesive for Precise Control of Adhesion Strength. ACS Appl. Mater. Interfaces.

[51] Kim M., Chung H. (2017). Photo-responsive bio-inspired adhesives: Facile control of adhesion strength via a photocleavable crosslinker. Polym. Chem..

[52] Fawcett W.R. (2008). The ionic work function and its role in estimating absolute electrode potentials. Langmuir.

[53] Isse A.A., Gennaro A. (2010). Absolute potential of the standard hydrogen electrode and the problem of interconversion of potentials in different solvents. J. Phys. Chem. B.

