# Supplementary material for: Visible-Light-Curable Solvent-Free Acrylic Pressure-Sensitive Adhesives via Photoredox-Mediated Radical Polymerization
Source: Molecules. 2021 Jan 13;26(2):385. doi: 10.3390/molecules26020385 (PMC7828379; doi:10.3390/molecules26020385)
Supplement: Supplementary file 1 [file molecules-26-00385-s001.pdf]

Supplementary material

Visible-Light-Curable Solvent-Free Acrylic Pressure-Sensitive Adhesives via Photoredox-Mediated Radical Polymerization

Jong-Ho Back [1], Yonghwan Kwon [2,3], Hyun-Joong Kim [4], Youngchang Yu [1,*], Wonjoo Lee [1,*] and Min Sang Kwon [2,*]

1 Center for Advanced Specialty Chemicals, Korea Research Institute of Chemical Technology, Ulsan 44412, Korea; beak1231@krict.re.kr

2 Department of Materials Science and Engineering, Seoul National University, Seoul 08826, Korea; yhkwon@unist.ac.kr

3 Department of Materials Science and Engineering, Ulsan National Institute of Science and Technology (UNIST), Ulsan 44919, Korea

4 Department of Agriculture, Forestry and Bioresources, Research Institute of Agriculture and Life Sciences, College of Agriculture and Life Science, Seoul National University, Seoul 08826, Korea; hjokim@snu.ac.kr

* Correspondence: ycyu@krict.re.kr (Y.Y.); winston@krict.re.kr (W.L.); minsang@snu.ac.kr (M.S.K.)

**Table of Content**	**Page**
1. Bulk polymerization (supporting)	S3
2. Non-consumable PC (UV/Vis spectrum)	S4
3. Remaining DBM% after bulk polymerization (GC)	S5
4. Manufacturing process and mechanism of solvent-free acrylic PSA	S6
5. Results with 50 ppm 4DP-IPN and 5 mol% DBM	S7
6. UV/Vis spectrum of PSA prepared through UV photoinitiator	S8
7. UV/Vis spectrum and PL intensity of 4DP-IPN	S9
8. Calculation of conversion at film curing (FT-IR)	S10
9. Method for evaluation of gel content	S11
10. Method for evaluation of adhesive properties	S12
11. Lap shear strength	S13
12. Characterization of representative sample	S14
13. Calibration curve of size-exclusion chromatography	S15
14. Reproducibility	S16
15. DFT calculation	S18
References	S20

1. Bulk polymerization (supporting)

Table S1. The Bulk polymerization results; α = conversion of bulk polymerization determined gravimetrically; M_n and Đ represents number average molecular weight and dispersity, respectively, which were characterized by size exclusion chromatography. Detail condition was same with the Table 1 in the manuscript.

Entry	α-Haloester	[M][a]:[4DP-IPN]:[a-Haloester]	Time (s)	α (%)	M_n (kg/mol)	Đ
S1	DBM	100:0.005:0.05	20	13.5	369	2.19
S2	DBM	100:0.005:0.05	10	4.67	304	2.52
S3	DBM	100:0.005:0.5	10	18.8	189	3.04
S4	DBM	100:0.005:5	10	31.9	84	2.60
S5	DBM	100:0.0002:0.1	10	4.24	446	2.09
S6	DBM	100:0.001:0	450	10.4	989	1.79
S7	DBM	100:0.001:0.05	10	3.46	613	2.05
S8	DBM	100:0.001:0.5	10	16.6	367	2.56
S9	DBM	100:0.001:5	10	34.8	218	2.52

[a]Comprising n-butyl acrylate (BA), isobornyl acrylate (IBOA), and 4-hydroxybutyl acrylate (HBA) (Mole ratio was set as follows; [BA]:[IBOA]:[HBA] = 80:10:10).

2. Non-consumable PC (UV/Vis spectrum)

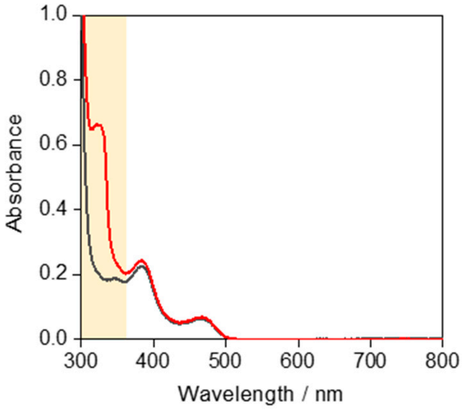

Figure S1. UV/Vis spectrum before and after bulk polymerization with 2 ppm of 4DP-IPN and 0.1 mol% of DBM (gray: before bulk polymerization, and red: after bulk polymerization).

UV-Vis spectrum after bulk polymerization was similar with that before bulk polymerization, which suggested that 4DP-IPN was not consumed during bulk polymerization. The difference in yellow box of Figure S1 might be resulted from DBM.

3. Remaining DBM% after bulk polymerization (GC)

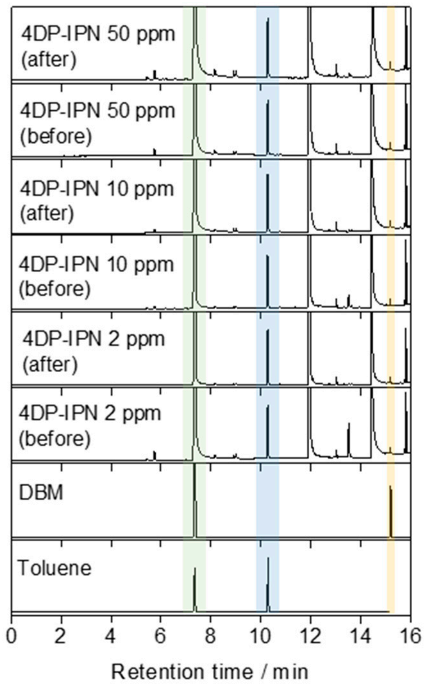

4DP-IPN content	Bulk polymerization	$I_{Toluene}$	I_{DBM}	$I_{DBM}/I_{Toluene}$	Consumed DBM	Residual DBM
2 ppm	Before	124.98	7.43	0.059434	0.2%	99.8%
	After	402.32	23.87	0.059331		
10 ppm	Before	244.84	19.46	0.079472	8.1%	91.9%
	After	177.55	12.97	0.073054		
50 ppm	Before	153.59	12.87	0.083780	11.8%	88.2%
	After	102.93	7.61	0.073891		

Figure S2. Gas chromatography results for evaluation of remaining DBM after bulk polymerization. Temperature step; 1st step: 35-130°C (10°C/min) and 2nd step: 130-325°C (30°C/min)). Retention time; ethyl acetate (solvent, green box): 7.352 min, toluene (internal standard, blue box): 10.297 min, DBM (yellow box): 15.219 min. DBM content was fixed as 0.1 mol%.

The residual DBM content was calculated by measuring the amount of DBM consumed during the bulk polymerization. In order to quantitatively evaluate consumed DBM, gas chromatography was employed and toluene was used as internal standard.

4. Manufacturing process and mechanism of solvent-free acrylic PSA

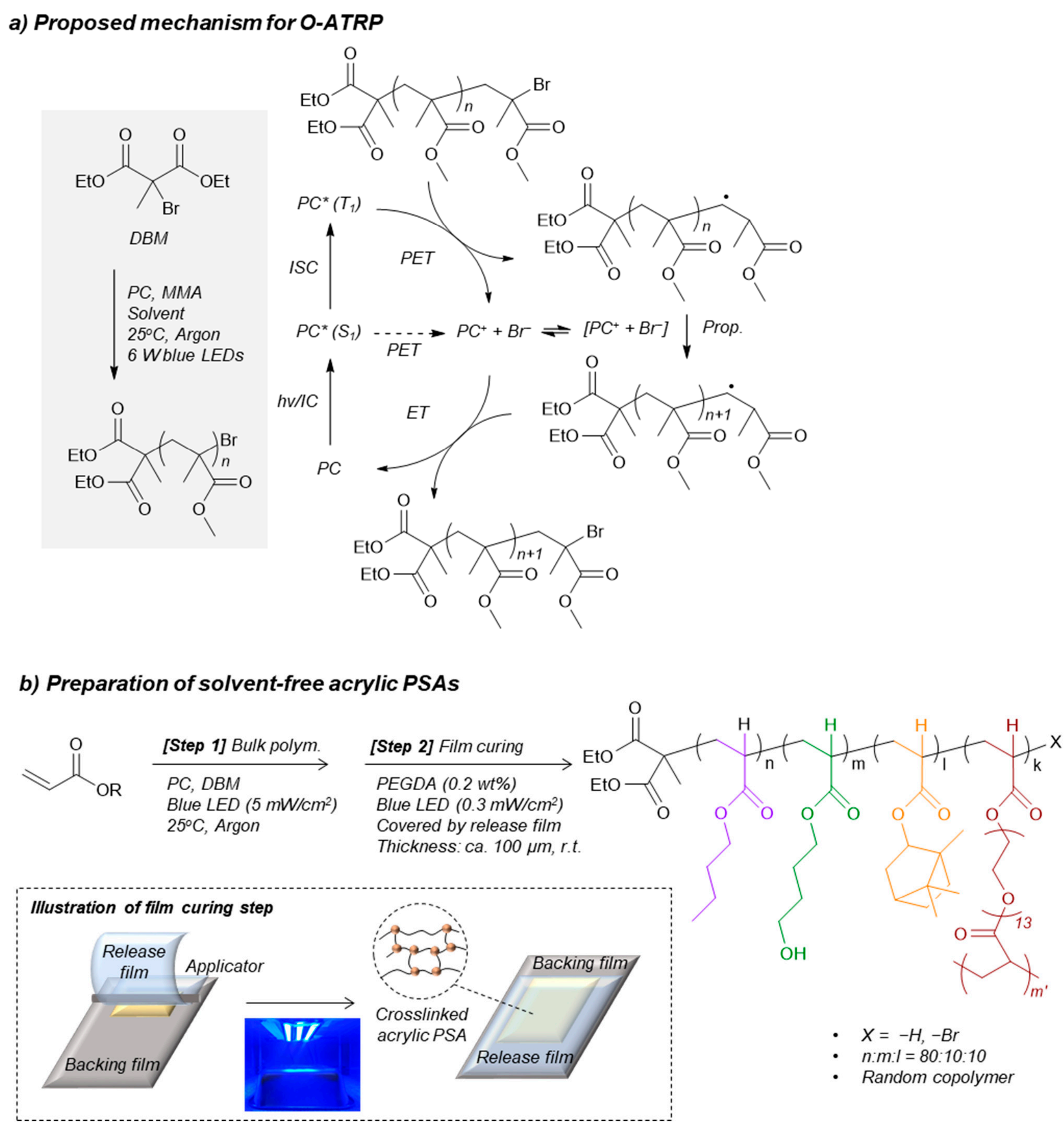

Figure S3. a) Mechanism of diethyl 2-bromo-2-methylmalonate (DBM)-initiated O-atom transfer radical polymerization (O-ATRP) proposed by Kwon et al. [24]. b) Manufacturing process of solvent-free acrylic PSA: Bulk polymerization (upper) and film curing (bottom).

5. Results with 50 ppm 4DP-IPN and 5 mol% DBM

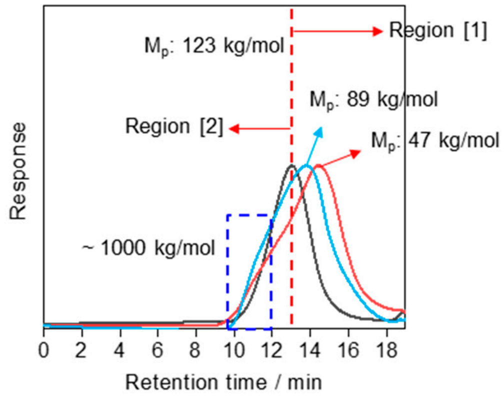

Bulk polymerization	$I_{Toluene}$	I_{DBM}	$I_{DBM}/I_{Toluene}$	Consumed DBM	Residual DBM
Before	392.90	1321.57	3.363672	7.9%	92.1%
After	153.19	474.46	3.097233		

Figure S4. Bulk polymerization results with 5 mol% of DBM and 50 ppm of PC: (upper) size exclusion chromatography curve (M_P: molecular weight at peak of SEC curve, gray: before film curing, red: after film curing without PEGDA, and blue: after film curing with 0.2 wt% of PEGDA). (bottom) gas chromatography results for evaluation of remaining DBM.

Pre-polymer with 5 mol% of DBM and 50 ppm of PC exhibited low molecular weight due to large amount of initiator. Before film curing, i.e. right after bulk polymerization, M_n and M_P was 84 kg/mol and 123 kg/mol, respectively. Although the crosslinker (PEGDA) was added, crosslinking network was not formed and M_P was lowered as 89 kg/mol, which might be resulted from a lot of remaining DBM (92.1%, from table of Figure S4). After film curing, the portion of "Region 1" in SEC curve, i.e. polymer initiated by remaining DBM, was larger than that of "Region 2" that represented slightly crosslinked polymer or re-initiated polymer from living chain end. When the crosslinker was not added, the portion of "Region 1" was enlarged.

6. UV/Vis spectrum of PSA prepared through UV photoinitiator

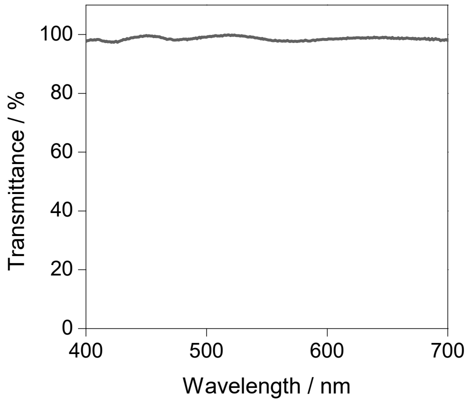

Figure S5. UV/Vis spectrum of PSA prepared through photoinitiator. 0.2 wt% of PEGDA was used as crosslinker. 0.3 wt% of Irgacure 184 was used as photoinitiator, and film was cured by 3 J/cm^2.

Transparency of PSA that prepared through photoinitiator was characterized by UV/Vis spectrum. After bulk polymerization (Entry 3, Table 1 of manuscript), crosslinker (PEGDA, 0.2 wt%) and photoinitiator (Irgacure 184, 0.3 wt%) were added, and then applied in a film form . The film was cured in the same way as shown in Figure S3, but UV (3 J/cm^2) was used instead of blue LED.

7. UV/Vis spectrum and PL intensity of 4DP-IPN

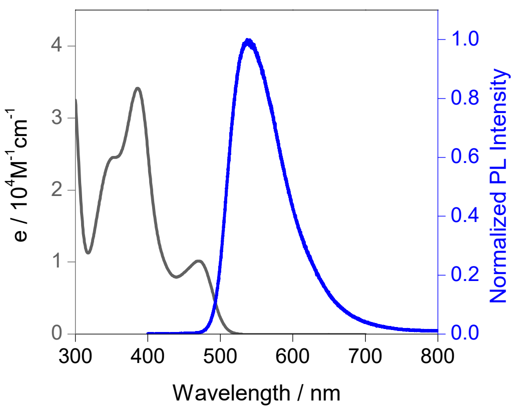

Figure S6. UV/Vis absorption spectrum (gray line) and photoluminescence (blue line) of 4DP-IPN that we used as visible-light PC. Gray line and blue line mean UV/Vis and PL spectrum, respectively. For characterization of UV/Vis absorption, photocatalyst was dissolved in acetonitrile (20 µm). For characterization of photoluminescence, photocatalyst was dissolved in dimethyl sulfoxide (20 µm) and λ_{ex} was 450 nm.

8. Calculation of conversion at film curing (FT-IR)

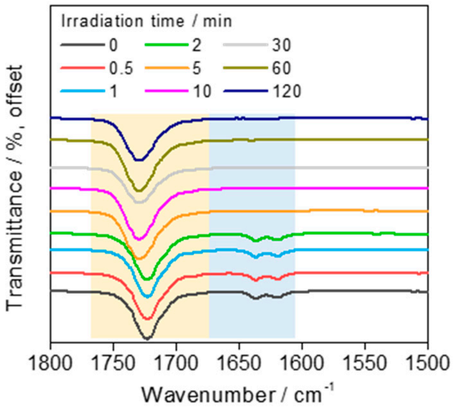

Figure S7. Calculation of conversion at film curing by using Fourier-transform infrared spectroscopy (FT-IR).

Conversion at film curing was calculated as follows;

$$Conversion\ (\%) = \frac{\frac{A_{O(C=C)}}{A_{o(C=O)}} - \frac{A_{t(C=C)}}{A_{t(C=O)}}}{\frac{A_{O(C=C)}}{A_{o(C=O)}}}$$

, where $A_{0(C=O)}$, $A_{t(C=O)}$, $A_{0(C=C)}$, and $A_{t(C=C)}$ mean peak area of carbonyl (1760-1660 cm^{-1}) at time 0, carbonyl at time t, C=C (1660-1600 cm^{-1}) at time 0, and C=C at time t.

9. Method for evaluation of gel content

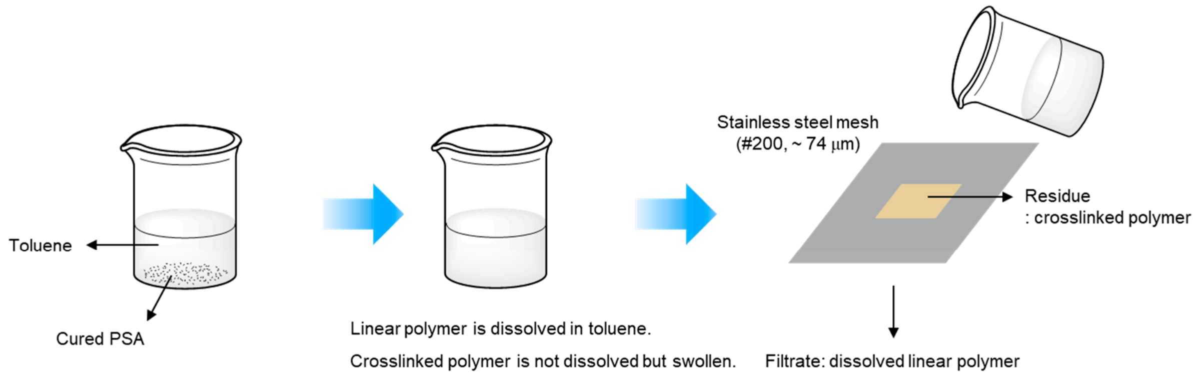

$$Gel\ content\ (\%) = \frac{W_r}{W_t} \times 100$$

W_r: weight of residue (crosslinked polymer)

W_t: total weight of cured PSA film

Figure S8. Method for evaluation and calculation of gel content.

10. Method for evaluation of adhesive properties

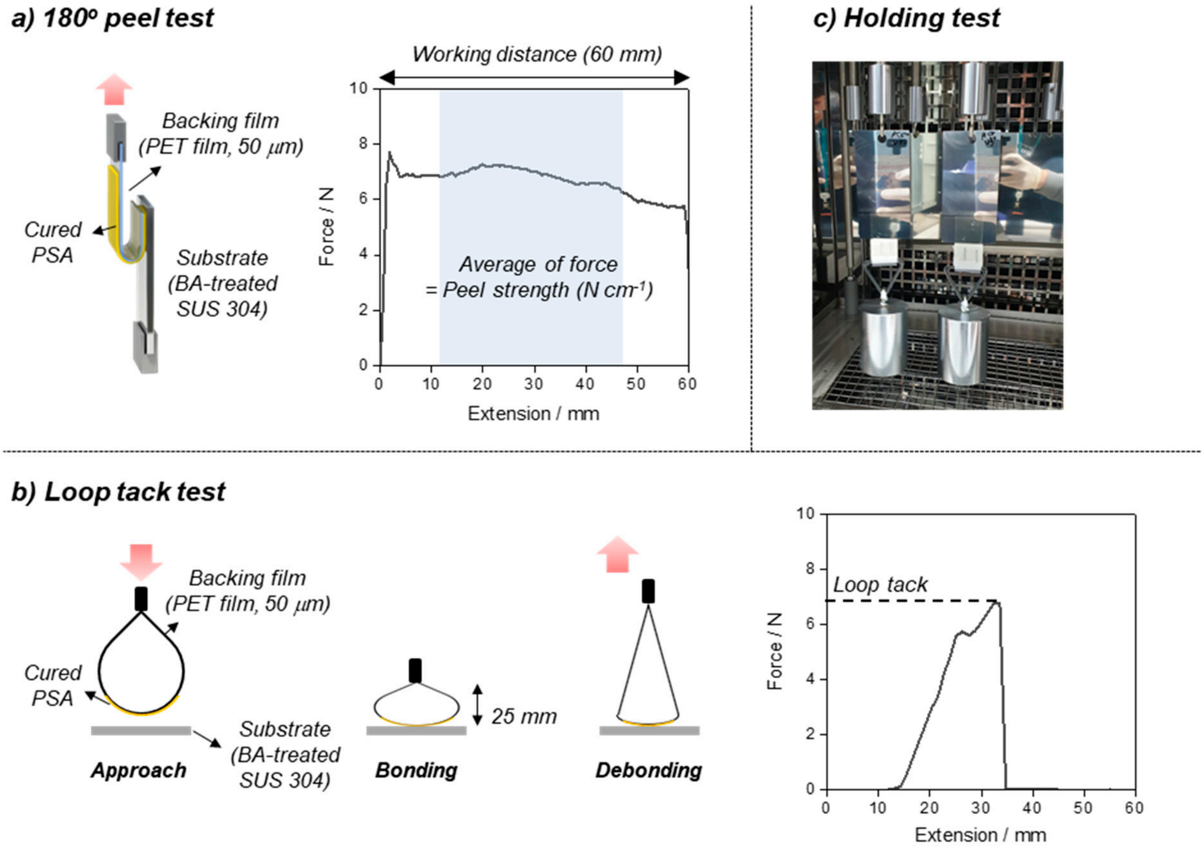

Figure S9. Method for evaluation of adhesive properties: (a) 180º peel test, (b) loop tack test, and (c) holding test.

11. Lap shear strength

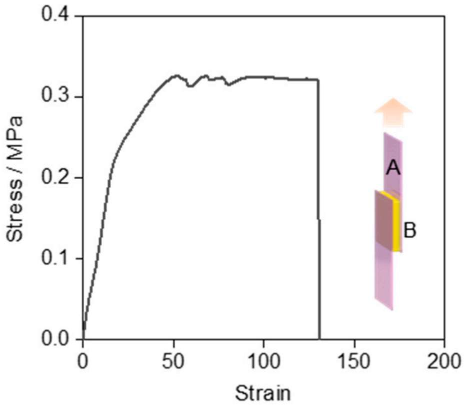

Entry	PC/DBM	Lap shear strength (MPa)
S2 (Table S1)	50 ppm/0.05 mol%	0.301 (±0.007)
S3 (Table S1)	50 ppm/0.5 mol%	0.329 (±0.007)
S4 (Table S1)	50 ppm/5 mol%	0.332 (±0.005)
5 (Table 1)	50 ppm/0.1 mol%	0.308 (±0.010)
6 (Table 1)	10 ppm/0.1 mol%	0.271 (±0.003)

Figure S10. Schematic illustration of single lap shear test and example of test result (upper, Entry S3, Table S1). A and B mean substrate (polyethylene terephthalate film, thickness: 50 μm) and PSA (thickness: 100 μm), respectively. Lap shear test results of entries S2, S3, S4, 5, and 6 are presented in the table.

Single lap shear test was conducted to evaluate structural characteristics of prepared PSAs. The cured PSA film was cut to a certain size (length: 50 mm, and width: 25 mm), and attached to substrate (polyethylene terephthalate film, length: 50 mm, width: 25 mm, and thickness: 0.05 mm). Specimens were attached by constant force (2 kg roller for twice) and stored at room temperature for 1 day. Universal testing machine (UTM 5982, Instron) was employed and strain-stress was obtained as shown in Figure S10. Maximum force of strain-stress curve mean lap shear strength and lap shear strength of entries S2, S3, S4, 5, and 6 was shown in Figure S10. There was no big difference in lap shear strength (≈ 0.3 MPa) among aforementioned entries, and the lap shear strength of prepared PSAs were comparable to those of photo-responsive acrylic PSAs in references (0.15~0.34 MPa) [39,50,51].

Lap shear strength means resistance to fracture of internal layer (Figure S10), whereas holding time we assessed means resistance to creep deformation (Figure 4). In addition, single lap shear test and holding test was conducted at different condition (single lap shear test: 25°C, holding test: 50°C) so that the results of lap shear strength could not correlate with the results of holding test.

12. Characterization of representative sample

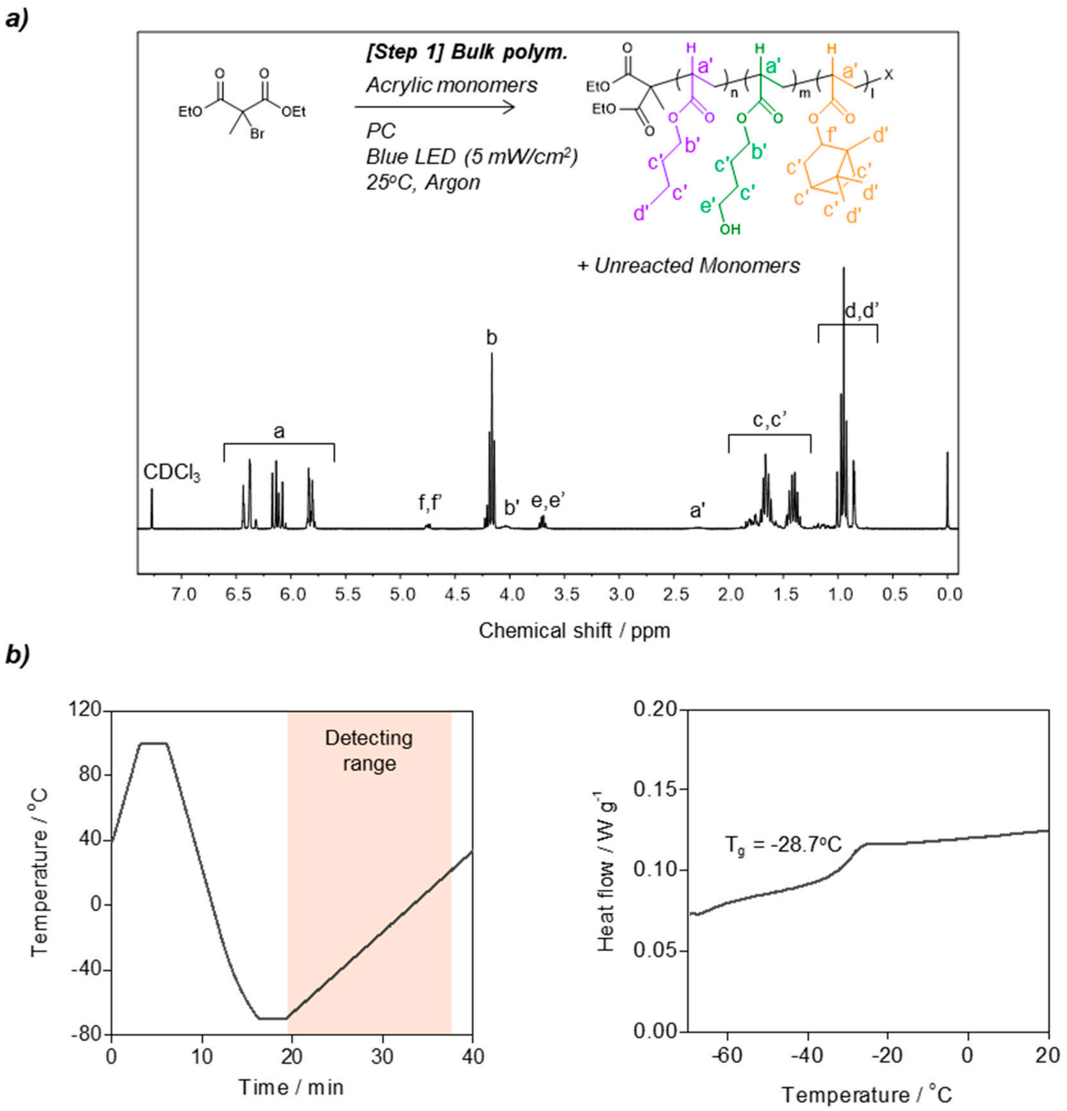

Figure S11. Characterization of Entry 6 in Table 1 (characterized after bulk polymerization). a) Nuclear magnetic resonance result (^{1}H-NMR, 300 MHz, Avance DPX-400, Bruker, solvent: $CDCl_3$). Symbol x in graph mean peak of x that was incorporated in unreacted monomer. Symbol x' in graph mean peak of x′ that was incorporated in polymer chain. b) Differential scanning calorimetry result (DSC, Q200, TA instrument, sample weight: 13 mg, sample was dried before characterization to remove unreacted monomers). Left graph presented time-temperature curve and detecting range. Right graph presented heat flow curve and T_g at detecting range.

After bulk polymerization, polymer chain dissolved in unreacted monomer was obtained, and its chemical structure was characterized by using ^{1}H-NMR (Figure S11a). Glass transition temperature (T_g) of synthesized polymer was obtained as -28.7°C (Figure S11b), which was low enough to be utilized as pressure-sensitive adhesive.

13. Calibration curve of size-exclusion chromatography

Retention time (min)	Molecular weight (g/mol)
12.096	364,000
12.7224	187,700
13.4064	107,100
14.4504	45,120
14.4504	45,120
15.0408	27,810
15.0552	27,811
15.4512	19,500
15.9624	12,980
16.2936	9,570
16.7184	6,320
16.7184	6,320
16.9848	4,750
17.3736	3,090
17.3952	3,091
17.784	1,920
18.1656	1,230
18.36	945
18.36	945
18.6768	580
19.0152	371
19.2672	370

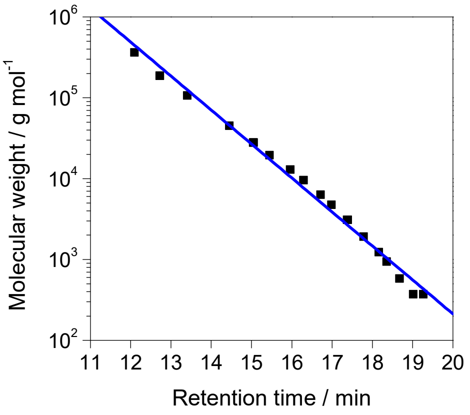

Figure S12. Calibration table (left) and curve (right) of size-exclusion chromatography. Linear polystyrene was used as standard.

14. Reproducibility

Table S2-1. Reproducibility result of Table 1. Detail information was same as presented in caption of Table 1. Samples marked with star in blue box were presented in the Table 1.

Entry	Time (s)	α (%)	M_n (kg/mol)	Đ
1 (Table 1)	> 8 h*	No separable polymers*		
	> 8 h	No separable polymers		
	> 8 h	No separable polymers		
2 (Table 1)	> 8 h*	No separable polymers*		
	> 8 h	No separable polymers		
	> 8 h	No separable polymers		
3 (Table 1)	15*	7.42*	873*	1.68*
	15	6.21	826	1.85
	15	6.88	814	1.87
4 (Table 1)	600*	11.2*	418*	2.34*
	500	11.3	558	2.36
	600	11.4	702	2.09
5 (Table 1)	10*	11.7*	289*	2.51*
	10	11.4	425	2.61
	10	8.02	377	2.53
6 (Table 1)	10	10.0	255	2.52
	10	6.14	611	2.17
	10	8.44	530	2.66
	10*	7.68*	512*	2.35*
7 (Table 1)	180*	7.76*	749*	1.83*
	180	9.41	1,018	1.84
	180	8.60	1,010	1.85
8 (Table 1)	100*	7.45*	514*	2.20*
	100	7.98	938	1.89
	100	6.95	739	2.10

Table S2-2. Reproducibility result of Table S1. Detail information was same as presented in caption of Table 1. Samples marked with star in blue box were presented in the Table S1.

Entry	Time (s)	α (%)	M_n (kg/mol)	Đ
S1 (Table S1)	20*	13.5*	369*	2.19*
	20	7.02	557	2.16
	20	10.83	477	2.50
S2 (Table S1)	10*	4.67*	304*	2.52*
	10	5.53	576	2.47
	10	5.06	536	2.59
S3 (Table S1)	10*	18.8*	189*	3.04*
	10	23.7	220	3.07
	10	17.9	226	3.15
S4 (Table S1)	10*	31.9*	84*	2.60*
	10	42.2	132	3.60
	10	41.8	136	3.30
S5 (Table S1)	10*	4.24*	446*	2.09*
	10	2.80	606	1.97
	10	2.98	680	1.78
S6 (Table S1)	450	10.5	994	1.76
	450	10.1	943	1.91
	450*	10.4*	989*	1.79*
S7 (Table S1)	10*	3.46*	613*	2.05*
	10	3.60	589	1.87
	10	3.54	523	2.04
S8 (Table S1)	10	16.2	357	2.36
	10*	16.6*	367*	2.56*
	10	18.2	392	2.53
S9 (Table S1)	10*	34.8*	218*	2.52*
	10	34.1	212	2.72
	10	36.9	205	2.90

15. DFT calculations

Density functional theory (DFT) and time-dependent (TD) DFT calculations were performed with the B3LYP functional and 6–311G* basis set as all implemented in the Gaussian16 program package. The geometries (S_0) optimization and single point energies were calculated in ethyl acetate solution employing polarizable continuum model (PCM). In all calculations, frequency calculations were accompanied both to verify that geometries were true minima and to obtain free energies at 298 K. In the case of PC, oxidation potentials were calculated by

$$\Delta G_{298,calc.}{}^{\circ}(PC^{\bullet+} / PC) = G_{298,PC^{\bullet+}}{}^{\circ} - G_{298,PC}{}^{\circ} \quad (1)$$

$$E_{298,calc}{}^{\circ} = -\frac{\Delta G_{298,calc}{}^{\circ}}{n_e F} \quad (2)$$

Subsequently, excited state redox potentials, $E_{ox,calc.}{}^{*}$, also were obtained from adiabatic transition energy of triplet in ethyl acetate solution, E_{adia} [24]. In the case of α-haloesters, because the examined α-haloesters undergo concerted dissociative electron transfer, the calculation of reduction potential of α-haloesters were derived from

$$\Delta G_{298,calc}{}^{\circ}(R-X / R\cdot + X^{-}) = (G_{298,R\bullet}{}^{\circ} + G_{298,Br^{-}}{}^{\circ}) - G_{298,R-Br}{}^{\circ} \quad (3)$$

The obtained free energies have standard reference state of 298.15 K and 1 atm by default of Gaussian16 program. Considering our system was carried out in the solution (bulk polymerization), the free energies need to be corrected. However, unlike the oxidation of PC, only reduction of α-haloesters have one net mole change as R-X dissociation and the free energies of α-haloesters were corrected by 1.89 kcal/mol. All of calculated redox potentials were referenced to SCE by subtraction of 4.42 V (abs.potential to SHE) [52] and 0.241 V (vs SHE into SCE) [53]. Molecular orbital (MO) topologies and electrostatic surface potential (ESP) were obtained by employed in Gaussian09w and plotted with GaussView 6.0.

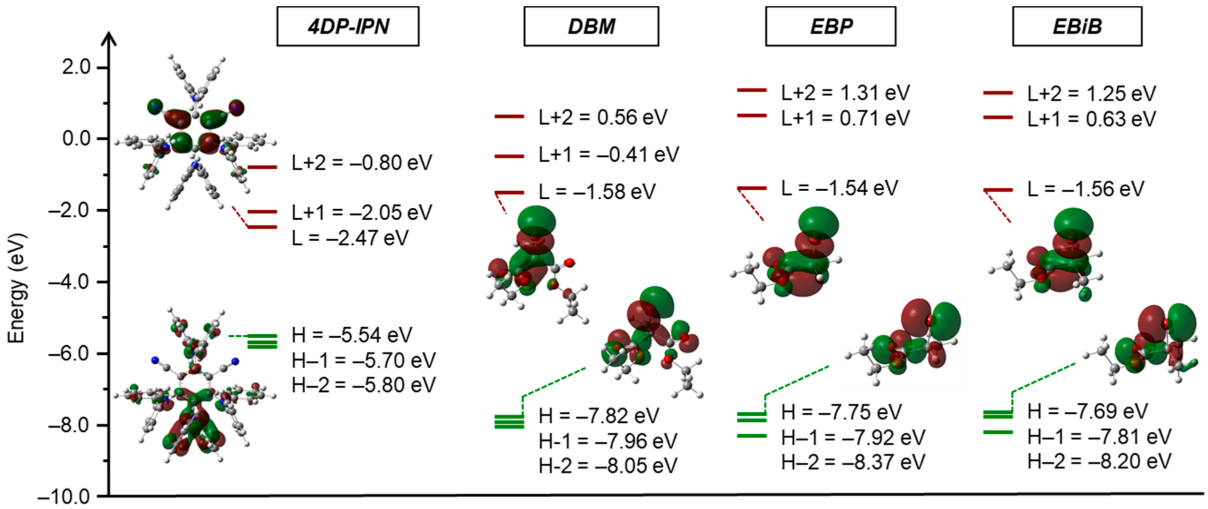

Figure S13. Density functional theory (DFT)-calculated molecular orbital (MO) diagrams and MO topologies of 4DP-IPN and α-haloesters; the time-dependent (TD)DFT calculations were performed with the B3LYP functional and 6–311G* basis set in ethyl acetate using the polarizable continuum model (PCM).
